# TGF-β protects osteosarcoma cells from chemotherapeutic cytotoxicity in a SDH/HIF1α dependent manner

**DOI:** 10.1186/s12885-021-08954-7

**Published:** 2021-11-11

**Authors:** Yangbo Xu, Yafei Li, Xiaofan Chen, Feifan Xiang, Yong Deng, Zhong Li, Daiqing Wei

**Affiliations:** 1 Department of Orthopaedics, The Affiliated Hospital of Southwest Medical University, Luzhou, 646000 Sichuan China; 2 Sichuan Provincial Laboratory of Orthopaedic Engineering, Luzhou, 646000 Sichuan China; 3 Department of Oncology, Luzhou People’s Hospital, Luzhou, 646000 Sichuan China; 4 Department of Pediatrics, Southwest Medical University, Luzhou, 646000 Sichuan China

**Keywords:** TGF-β, Chemoresistance, Succinate dehydrogenase, Osteosarcoma, Hypoxia-inducible factor 1α (HIF1α)

## Abstract

**Background:**

In the widespread adoption of chemotherapy, drug resistance has been the major obstacle to tumor elimination in cancer patients. Our aim was to explore the role of TGF-β in osteosarcoma-associated chemoresistance.

**Methods:**

We performed a cytotoxicity analysis of methotrexate (MTX) and cisplatin (CIS) in TGF-β-treated osteosarcoma cells. Then, the metabolite profile of the core metabolic energy pathways in Saos-2 and MG-63 cell extracts was analyzed by ^1^H-NMR. We detected the expression of succinate dehydrogenase (SDH), STAT1, and hypoxia-inducible factor 1α (HIF1α) in TGF-β-treated osteosarcoma cells and further tested the effects of these molecules on the cytotoxicity induced by chemotherapeutic agents. Using in vivo experiments, we examined the tumor growth and survival time of Saos-2-bearing mice treated with a combination of chemotherapeutic agents and a HIF1α inhibitor.

**Results:**

The metabolic analysis revealed enhanced succinate production in osteosarcoma cells after TGF-β treatment. We further found a decrease in SDH expression and an increase in HIF1α expression in TGF-β-treated osteosarcoma cells. Consistently, blockade of SDH efficiently enhanced the resistance of Saos-2 and MG-63 cells to MTX and CIS. Additionally, a HIF1α inhibitor significantly strengthened the anticancer efficacy of the chemotherapeutic drugs in mice with osteosarcoma cancer.

**Conclusion:**

Our study demonstrated that TGF-β attenuated the expression of SDH by reducing the transcription factor STAT1. The reduction in SDH then caused the upregulation of HIF1α, thereby rerouting glucose metabolism and aggravating chemoresistance in osteosarcoma cells. Linking tumor cell metabolism to the formation of chemotherapy resistance, our study may guide the development of additional treatments for osteosarcoma.

## Background

Osteosarcoma is the most prevalent malignant bone tumor, with a high occurrence in children and adolescents [1]. Surgery combined with chemotherapeutic agents is considered the major strategy for treatment. However, many patients with osteosarcoma still develop pulmonary metastases and disease relapse, resulting in a poor survival rate [2, 3]. Recently, the resistance of tumor cells to drugs such as methotrexate (MTX), cisplatin (CIS) and doxorubicin has emerged as an important barrier to the treatment of osteosarcoma [4]. Therefore, further research is warranted to better understand osteosarcoma chemoresistance and formulate efficacious therapeutic strategies.

Drug resistance, a multifaceted process, is attributed to a combination of factors comprising apoptosis induction, autophagy induction, cancer stem cell regulation, DNA damage and repair, and epigenetic regulation [5]. Emerging evidence has supported the role of tumor metabolism in promoting drug resistance [6, 7]. Cancer cells rewire their metabolism to satisfy the high demand for both energy and biosynthesis. In this context, glycolysis is given a high priority in cancer cells even under physiological oxygen conditions, which is named the “Warburg effect”. Components of the glycolytic pathway, such as glucose transporters and hexokinase-2, the first rate-limiting enzyme in the glycolytic pathway, are intimately linked to chemoresistance [8, 9]. These findings raise awareness of intervening tumor metabolism to combat drug resistance.

It has been established that transforming growth factor-β (TGF-β) may function as a significant contributor to drug resistance in several types of tumors [10–14]. TGF-β, a pleiotropic cytokine, plays a key role in regulating multiple biological processes, including cell proliferation, immune response and inflammation [15]. Reportedly, upregulation of TGF-β signaling has been found in erlotinib-resistant lung cancer cells [16]. Upon attaching to its receptors, TGF-β induces a series of events, among which the epithelial-mesenchymal transition (EMT) endows cancer cells with metastatic and invasive properties. Compelling reports have demonstrated that the EMT confers chemoresistance to cancer cells by increasing drug efflux pumps and antiapoptotic effects [17]. TGF-β has thus become a promising target in cancer therapy, and treatments targeting the TGF-β pathway, such as neutralizing antibodies and soluble TGF-β receptors, have been evaluated in preclinical tests and even clinical trials [18].

In the current study, we observed stronger TGF-β expression in chemoresistant osteosarcoma patients, and in vitro TGF-β treatment obviously strengthened the multidrug resistance of osteosarcoma cell lines. Mechanistically, we demonstrated that TGF-β caused a decrease in STAT1 to inhibit the metabolic enzyme succinate dehydrogenase (SDH), giving rise to succinate accumulation in osteosarcoma cells. We further elucidated that the increase in succinate level promoted the expression of hypoxia-inducible factor 1α (HIF-1α), and blockade of HIF1α expression in turn augmented the antitumor effects of MTX and CIS. These findings revealed the molecular basis of TGF-β implicated in the regulation of metabolic pathways and subsequent chemoresistance of osteosarcoma.

## Methods

### Cell lines and reagents

The human osteosarcoma cell lines Saos-2 and MG-63 were obtained from the Cell Bank of the Chinese Academy of Sciences (Shanghai, China) and cultured in Dulbecco’s modified Eagle’s medium (DMEM) with 10% fetal bovine serum (FBS) (Gibco). All cells were grown at 37 °C in an incubator with 5% CO_2_. MTX, CIS, succinate, and KC7F2 were purchased from Sigma-Aldrich (ST, USA). Recombinant human and mouse TGF-β1 proteins were purchased from Pepro Tech (Rocky Hill, NJ).

### Patients and specimens

This study was approved by the Ethics Committee of the Affiliated Hospital of Southwest Medical University. Written informed consent was obtained from all patients, and all methods were performed according to the Declaration of Helsinki. A total of 20 osteosarcoma patients were recruited and provided informed consent between January 2017 and January 2020. These patients were divided into chemosensitive and chemoresistant groups according to the Response Evaluation Criteria in Solid Tumors (RECIST). Then, osteosarcoma specimens were collected, fixed in formalin, and embedded in paraffin for further detection.

For 3D matrix gel culture, tumor tissues were minced and digested with collagenase (Sigma-Aldrich, MA, USA) followed by filtration (BIOFIL). After centrifugation and removal of the red blood cells, osteosarcoma cells were seeded into 3D matrix gels in DMEM with 10% FBS.

### Metabolic assessment of cells

Metabolic assessment of Saos-2 and MG-63 cells (1 × 10^7^ cells per sample) was performed by NMR as previously described [19, 20]. Briefly, after using a methanol–chloroform–water extraction method, the upper aqueous phase was lyophilized and then redissolved in 550 μl of phosphate buffer solution (60 mM K_2_HPO_4_/NaH_2_PO_4_, pH 7.4, and 99.9% D_2_O) [21]. A Bruker 600-MHz spectrometer was used for the ^1^H-NMR experiments at 277 K. Quantitative analysis of metabolites was performed using TopSpin (version 3.5) software. Metabolites were assigned according to published data. Metabolite concentrations were quantified per million cells, and mean cell metabolite concentrations (fold change) were then calculated.

### Metabolite quantification

Quantitative analysis of succinate and fumarate was conducted using succinate (succinic acid) and fumarate colorimetric assay kits (BioVision, SF, USA), respectively, under the supplied instructions.

### Cytotoxicity analysis

Cytotoxicity analysis was performed using a FITC-Annexin V/PE-PI apoptosis detection kit (BD, NJ, USA) according to the manufacturer’s instructions. Briefly, after treatment with 75 mM MTX or 40 μM CIS for 48 h, osteosarcoma cells were stained with FITC-Annexin V and PE-PI staining solution. Apoptosis was detected on a C6 flow cytometer (BD, NJ, USA). Each experiment was repeated independently in triplicate.

### SiRNA silencing

Transfection of siRNAs was performed with Lipofectamine 8000 (Beyotime, Beijing, China) according to the suppliers’ protocol. The relevant siRNA sequences were as follows: SDHD-si#1: 5′-GCTCACAATAAGGAAGAAATA-3′; SDHD-si#2: 5′-GCCGAGCTCTGTTGCT TCGAA-3′; STAT1-si#1: 5′-CTGGAAGATTTACAAGATGAA-3′; STAT1-si#2: 5′-CCCTGAAGTATCTGTATCCAA-3′.

### Real-time PCR

Total RNA was extracted with TRIzol (Invitrogen, CA, USA) and transcribed into cDNA by using a high capacity cDNA reverse transcription kit (Applied Biosystems, CA, USA). PCR was performed on an ABI StepOne Plus (Applied Biosystems, MA, USA). The primer sequences were as follows: STAT1, 5′-CAGCTTGACTCAAAATTCCTGGA-3′ (sense) and 5′-TGAAGATTACGCTTGC TTTTCCT-3′ (antisense); SDHD, 5′-CATCTCTCCACTGGACTAGCG-3′ (sense) and 5′-TCCATCGCAGAGCAAGGATTC-3′ (antisense); and GAPDH, 5′ -GGAGCGA GATCCCTCCAAAAT-3′ (sense) and 5′-GGCTGTTGTCATACTTCT CATGG-3′ (antisense). The results were confirmed by at least three independent experiments.

### Western blot analysis

Cells were collected and lysed in NP40 solution. Then, the protein samples were run on an SDS–PAGE gel and transferred to nitrocellulose membranes; these membranes were blocked in 5% bovine serum albumin (BSA) and probed with primary antibodies against β-actin (Cell Signaling, Cat No. 3700; 1:1000); SDHD (Abcam, ab189945; 1:500); phospho-Jak1 (Cell Signaling, Cat No. 74129; 1:1000); Jak1 (Cell Signaling, Cat No. 50996; 1:1000); phospho-Jak2 (Cell Signaling, Cat No. 3776; 1:1000); Jak2 (Cell Signaling, Cat No. 3230; 1:1000); phospho-Stat1 (Cell Signaling, Cat No. 9167; 1:1000); Stat1 (Cell Signaling, Cat No. 9172; 1:1000); phospho-Stat2 (Cell Signaling, Cat No. 8410; 1:1000); Stat2 (Cell Signaling, 919, Cat No. 9172; Stat1:1000); phospho-Stat2 (Cell Signaling, Cat No. 88410; 1000); Stat3 (Cell Signaling, Cat No. 9139; 1:1000); HIF1α (Abcam, ab179483; 1:1000). Incubation with secondary antibodies conjugated to horseradish peroxidase was performed for 1 hour at room temperature. The proteins detected were visualized by enhanced chemiluminescence (Thermo Fisher, MA, USA).

### Immunohistochemical and immunofluorescence staining

Tumor tissues from patients were fixed in 37% formalin and embedded in paraffin. After retrieval of antigens, sections were stained with primary antibodies against TGF-β1 (Abcam, ab215715; 1:500), SDHD (Abcam, ab189945; 1:200), and phospho-Stat1 (Cell Signaling, Cat No. 9167; 1:500) at 4 °C overnight. Immunohistochemical staining was performed using a DAB Horseradish Peroxidase Color Development Kit (Beyotime, Shanghai, China) according to the supplied protocol. In brief, tissue slides were stained with HRP-conjugated secondary antibodies (Thermo, Cat No. G-21234, 1:1000) for 1 h at room temperature and stained with hematoxylin (Solarbio, Beijing, China). For immunofluorescent staining, tissue slides were incubated with secondary antibody followed by incubation with DAPI (Solarbio, Beijing, China). The intensity of the immunostaining was analyzed by ImageJ 9.0 software. In each sample, 25 regions were pictured randomly, and the mean expression value of 25 regions was determined as the relative expression value of protein in this sample. In each group, including the CR and CS groups, five tumor tissues were collected and analyzed for difference analysis.

### Animal experiments

NSG mice (4–6 weeks old) were purchased from HFK Bioscience Company (Beijing, China) and maintained under pathogen-free conditions. For tumor growth analysis, 2 × 10^6^ Saos-2 or MG-63 cells were subcutaneously injected into NSG mice. Then, these mice were randomized into different groups 10 days after inoculation and treated with or without TGF-β (20 μg/kg), MTX (5 mg/kg), CIS (1 mg/kg), or KC7F2 (10 mg/kg) twice a week for 14 days. The mice in the control groups received an equal volume of saline. Tumor growth (*n* = 6 in each group) was examined every other day, and the survival of mice (n = 6 in each group) was recorded. Tumor volume was calculated using the formula: tumor volume = length×width^2^/2.

For tumorigenesis analysis, NSG mice received subcutaneous injections of 2 × 10^5^ Saos-2 or MG-63 cells. Ten days after inoculation, these mice were treated with or without TGF-β (20 μg/kg) or KC7F2 (10 mg/kg) twice a week for 14 days. Tumorigenesis was calculated 20 days after injection (*n* = 6 in each group). The number of mice with tumor formation in a total of 6 mice was determined as the tumorigenesis rate. The above experimental procedures were approved by the Ethics Committee of the Affiliated Hospital of Southwest Medical University, according to the National Institutes of Health Guide for the Care and Use of Laboratory Animals. All animal studies were conducted in accordance with the Public Health Service Policy and complied with the ARRIVE guidelines for the humane use and care of animals.

### Statistical analysis

All experiments were independently performed in triplicate. The results are presented as the mean ± SEM and were analyzed by Student’s t-test or one-way ANOVA. The survival rates were determined by Kaplan-Meier survival analysis. A *P* value < 0.05 was considered statistically significant. The analysis was performed using GraphPad 8.0 software.

## Results

### TGF-β promoted osteosarcoma chemoresistance

**Fig. 1:**
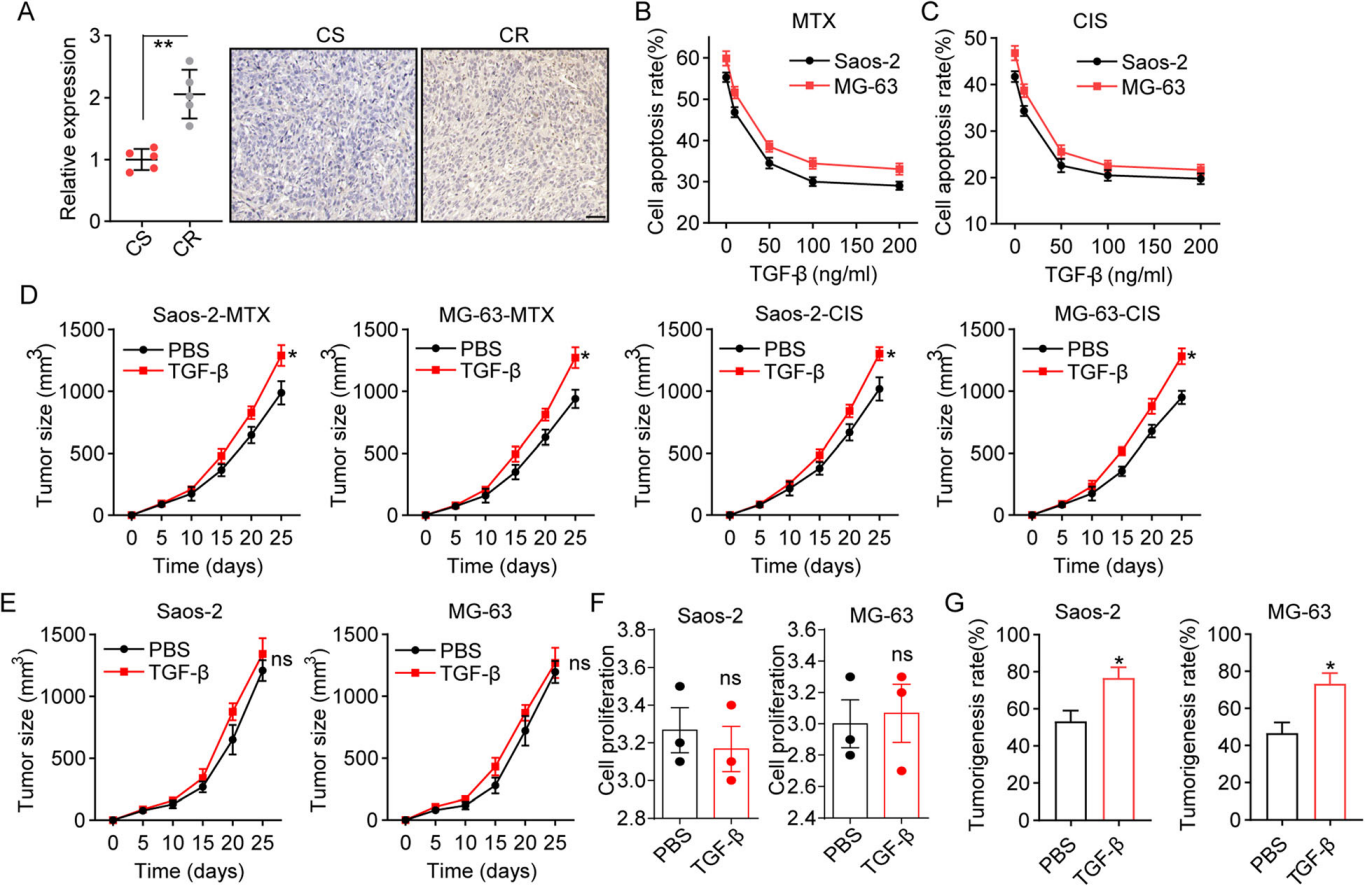
TGF-β promoted osteosarcoma chemo-resistance. **A** Immunohistochemistry of TGF-β in tumor tissues from chemoresistant (CR) and chemosensitive (CS) patients. The scale bar is 100 μm. **B** Saos-2 and MG-63 cells were treated with MTX (75 mM) plus different concentrations of TGF-β for 48 h. Cell apoptosis was determined by flow cytometry. **C** The same as B except that Saos-2 and MG-63 cells were treated with CIS (40 μM) plus different concentrations of TGF-β. **D** The NSG mice with Saos-2 and MG-63 osteosarcoma were treated with MTX (5 mg/kg)/CIS (1 mg/kg) plus TGF-β (20 μg/kg). The tumor growth was measured. **E** The NSG mice with Saos-2 and MG-63 osteosarcoma were treated with TGF-β (20 μg/kg). The tumor growth was measured. **F** relative cell proliferation of Saos-2 and MG-63 cells treated with PBS or TGF-β (50 ng/ml)**.** G Tumorigenicity of Saos-2 and MG-63 cells after treatment with TGF-β (20 μg/kg). MTX, methotrexate; CIS, cisplatin

Compelling reports have indicated that tumor tissues with increasing TGF-β expression exhibit enhanced migratory features and poor prognosis [22]. Our study further explored the role of TGF-β in osteosarcoma progression, including drug resistance and tumorigenicity. To do this, we isolated tumor tissues from chemoresistant/sensitive osteosarcoma patients and examined the expression of TGF-β in tumor tissues. Notably, tumor tissues isolated from chemoresistant patients exhibited elevated TGF-β expression compared to the chemosensitive group (Fig. 1A), suggesting the potential role of TGF-β in the chemoresistant development of osteosarcoma. Given the limited sample size, we further performed cytotoxicity analysis in TGF-β-treated Saos-2 and MG-63 osteosarcoma cell lines. As a result, TGF-β treatment significantly strengthened the resistance of Saos-2/MG-63 cells to chemotherapeutic MTX (Fig. 1B) and CIS (Fig. 1C). Similar results were observed in TGF-β-treated Saos-2/MG-63-bearing mice (Fig. 1D). These results suggested that TGF-β could facilitate chemoresistance in osteosarcoma cells. Subsequently, we further assessed the influence of TGF-β on tumor growth and cell proliferation. Although no significant difference was observed in the tumor volumes of Saos-2/MG-63-bearing mice (Fig. 1E) or cell proliferation (Fig. 1F), Saos-2 and MG-63 cells treated with TGF-β exhibited a strengthened tumorigenesis capability (Fig. 1G), indicating of the potential relationship between TGF-β and stem-associated transcription factors in osteosarcoma. Together, these results suggested that TGF-β can promote osteosarcoma chemoresistance, resulting in poor outcomes in patients.

### TGF-β suppressed succinate dehydrogenase to promote chemoresistance

**Fig. 2:**
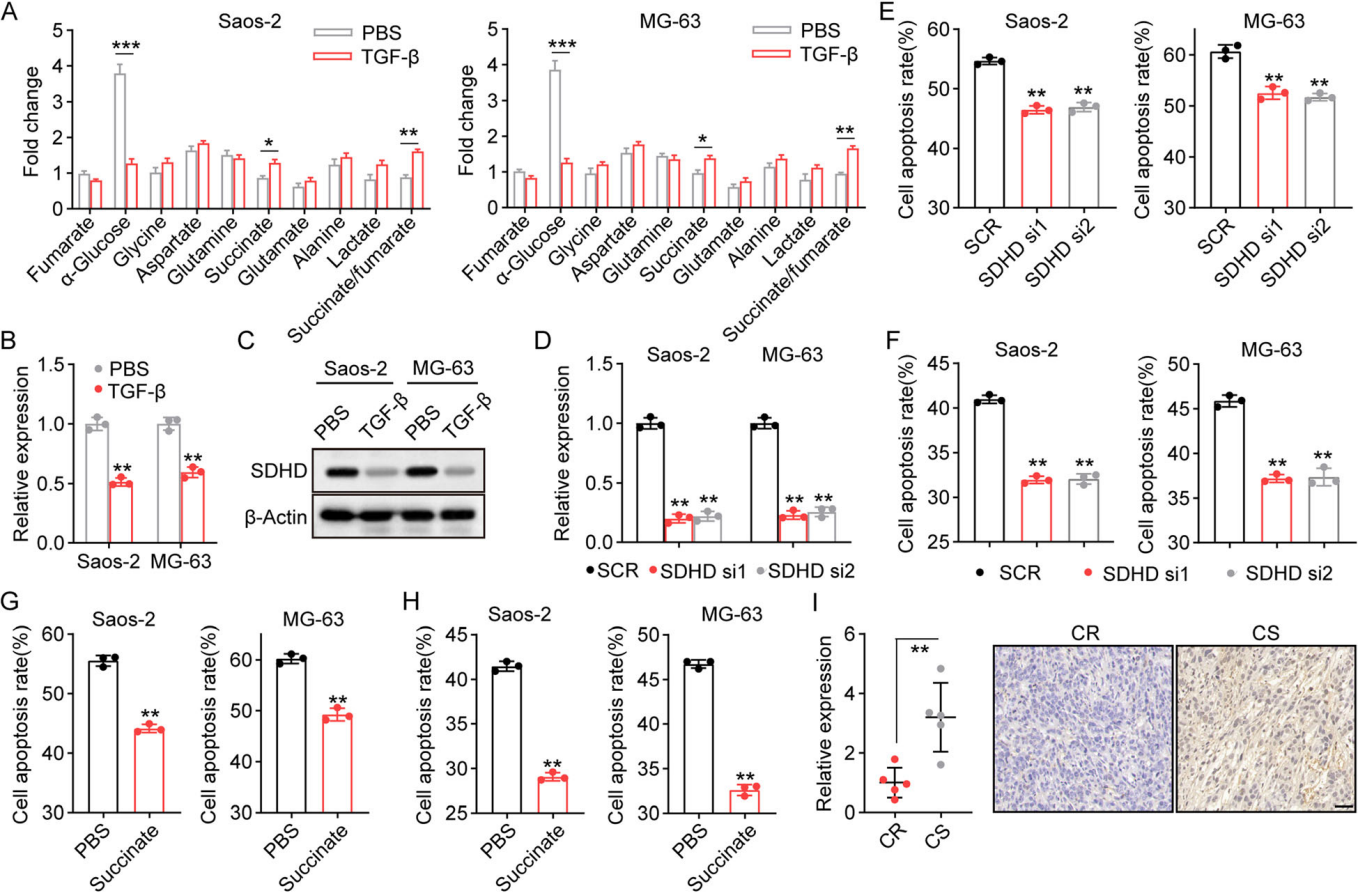
TGF-β suppressed succinat dehydrogenase to promote chemo-resistance. **A** Metabolite quantification by ^1^H-NMR. Results show the metabolite ratio in Saos-2/MG-63 cells after treatment with TGF-β (50 ng/ml). **B, C** The expression of SDHD in Saos-2 and MG-63 cells after treatment with TGF-β (50 ng/ml) was determined by quantitative real-time PCR (**B**) and western blot (**C**). **D** The knockdown of SDHD in Saos-2 and MG-63 cells was analyzed by quantitative real-time PCR. **E, F** Saos-2 and MG-63 cells were transfected with scramble siRNA (SCR), SDHD siRNA1 or SDHD siRNA2, and treated with 75 mM MTX (**E**) or 40 μM CIS (**F**) for 48 h. Scramble-Saos-2/MG-63 cells were used as control. Cell apoptosis was determined by flow cytometry. **G** Saos-2 and MG-63 cells were treated with MTX (75 mM) plus succinate (20 mM) for 48 h. Cell apoptosis was determined by flow cytometry. **H** The same as G, except that Saos-2 and MG-63 cells were treated with CIS (40 μM) plus succinate for 48 h. **I** Immunohistochemistry of SDHD in tumor tissues from chemoresistant (CR) and chemosensitive (CS) patients. The scale bar is 50 μm. MTX, methotrexate; CIS, cisplatin

Alteration of energy metabolism is a biological fingerprint of tumor cells, and enhanced lactate production (caused by glycolysis) correlates with drug resistance in several tumor types [23]. Here, a metabolite profile of the core energetic routes was analyzed by ^1^H-NMR in Saos-2/MG-63 cell extracts. After TGF-β treatment, Saos-2/MG-63 cells revealed obviously higher consumption of glucose and increased production of lactate (Fig. 2A), suggesting that tumor cells might enhance glycolysis by preferential conversion of pyruvate to lactate instead of oxidation. In addition, enhanced succinate production and an increased succinate/fumarate ratio were observed in Saos-2/MG-63 cells treated with TGF-β, as shown in Fig. 2A. These results prompted us to speculate that TGF-β might affect SDH metabolism, resulting in the accumulation of succinate and suppression of oxidation in the tricarboxylic acid cycle. In that case, the expression of SDHD, a submit of SDH, was examined. Intriguingly, suppression of SDHD expression was observed at the mRNA (Fig. 2B) and protein (Fig. 2C) levels in TGF-β-treated Saos-2/MG-63 cells. To further confirm the role of SDH in osteosarcoma progression, siRNA inference was conducted to suppress the expression of SDHD (Fig. 2D). Blockade of SDHD obviously strengthened the resistance of Saos-2/MG-63 cells to MTX and CIS (Fig. 2E and F). In addition, the addition of succinate to suppress oxidation also promoted MTX/CIS resistance in osteosarcoma cells (Fig. 2G and H). Consistently, suppression of SDHD was observed in chemoresistant tumor tissues obtained from patients (Fig. 2I). Together, these results suggested that TGF-β can suppress SDH to promote chemoresistance in osteosarcoma.

### TGF-β suppressed STAT1 to decrease SDH expression

**Fig. 3:**
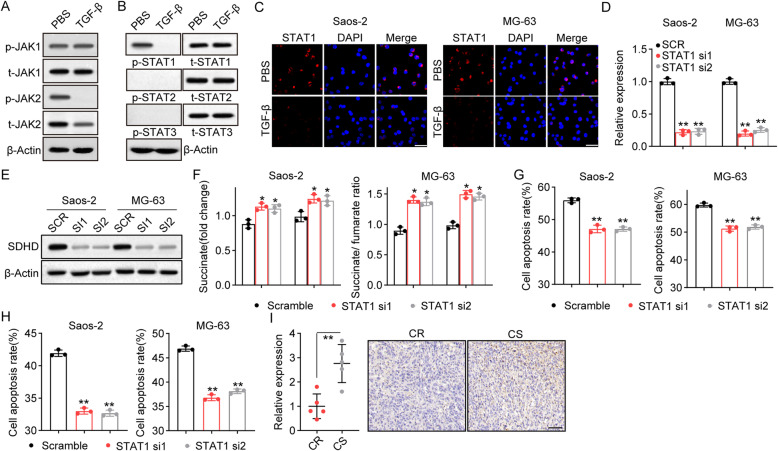
TGF-β suppressed STAT1 to decrease SDH expression. **A** Saos-2 cells were treated with TGF-β (50 ng/ml) for 48 h and then the expression of phosphorylated JAK1, JAK1, phosphorylated JAK2 and JAK2 was determined by western blot. **B** Saos-2 cells were treated with TGF-β (50 ng/ml) for 48 h and then the expression of phosphorylated STAT1, STAT1, phosphorylated STAT2, STAT2, phosphorylated STAT3 and STAT3 was determined by western blot. **C** Immunofluorescence staining of phosphorylated STAT1 in Saos-2 and MG-63 cells with or without TGF-β (50 ng/ml) treatment. The scale bar is 50 μm. **D** The knockdown of STAT1 in Saos-2 and MG-63 cells was analyzed by quantitative real-time PCR. **E** Saos-2 or MG-63 cells were transfected with scramble siRNA (SCR), STAT1 siRNA1 or STAT1 siRNA2, and the expression of SDHD was determined by western blot. **F** Metabolite quantification in scramble- or shSTAT1-Saos-2/MG-63 cells treated with TGF-β (50 ng/ml). G, H Scramble- or shSTAT1-Saos-2/MG-63 cells were treated with 75 mM MTX (**G**) or 40 μM CIS (**H**) for 48 h (containing 50 ng/ml TGF-β in culture medium). Cell apoptosis was determined by flow cytometry. **I** Immunohistochemistry of phosphorylated STAT1 in tumor tissues from chemoresistant (CR) and chemosensitive (CS) patients. The scale bar is 100 μm. MTX, methotrexate; CIS, cisplatin

Current studies provide evidence to suggest that TGF-β can regulate JAK/STAT-associated signaling pathways to promote cancer progression [24]. To further clarify the mechanism in SDH-associated osteosarcoma progression, the expression of JAK1, JAK2, STAT1, STAT2, and STAT3 was examined by western blotting. Notably, reduced expression of phosphorylated JAK2 and STAT1 was observed in TGF-β-treated Saos-2 cells (Fig. 3A and B). In addition, reduced nuclear entry of STAT1 in TGF-β-treated Saos-2 and MG-63 cells was found by immunofluorescence staining (Fig. 3C), suggesting that TGF-β suppressed STAT1 activation in osteosarcoma cells. Next, siRNA interference was performed to suppress STAT1 expression in Saos-2 and MG-63 cells (Fig. 3D). Subsequently, silencing STAT1 downregulated the expression of SDHD in Saos-2 and MG-63 cells (Fig. 3E). Enhanced succinate production and an increased succinate/fumarate ratio were found in STAT1-silenced Saos-2 and MG-63 cells (Fig. 3F). These results indicated that TGF-β suppressed STAT1 to downregulate SDH activity. Subsequently, we observed similar chemoresistance to MTX (Fig. 3G) and CIS (Fig. 3H) in STAT1-silenced Saos-2 and MG-63 cells, suggesting that STAT1 signals were involved in TGF-β-associated chemoresistance. Consistently, STAT1 levels were reduced in chemoresistant tumor tissues from osteosarcoma patients (Fig. 3I). These results suggested that TGF-β suppressed STAT1 signaling to downregulate SDH metabolism.

### Metabolic succinate facilitated osteosarcoma chemoresistance in a HIF1α-dependent manner

**Fig. 4:**
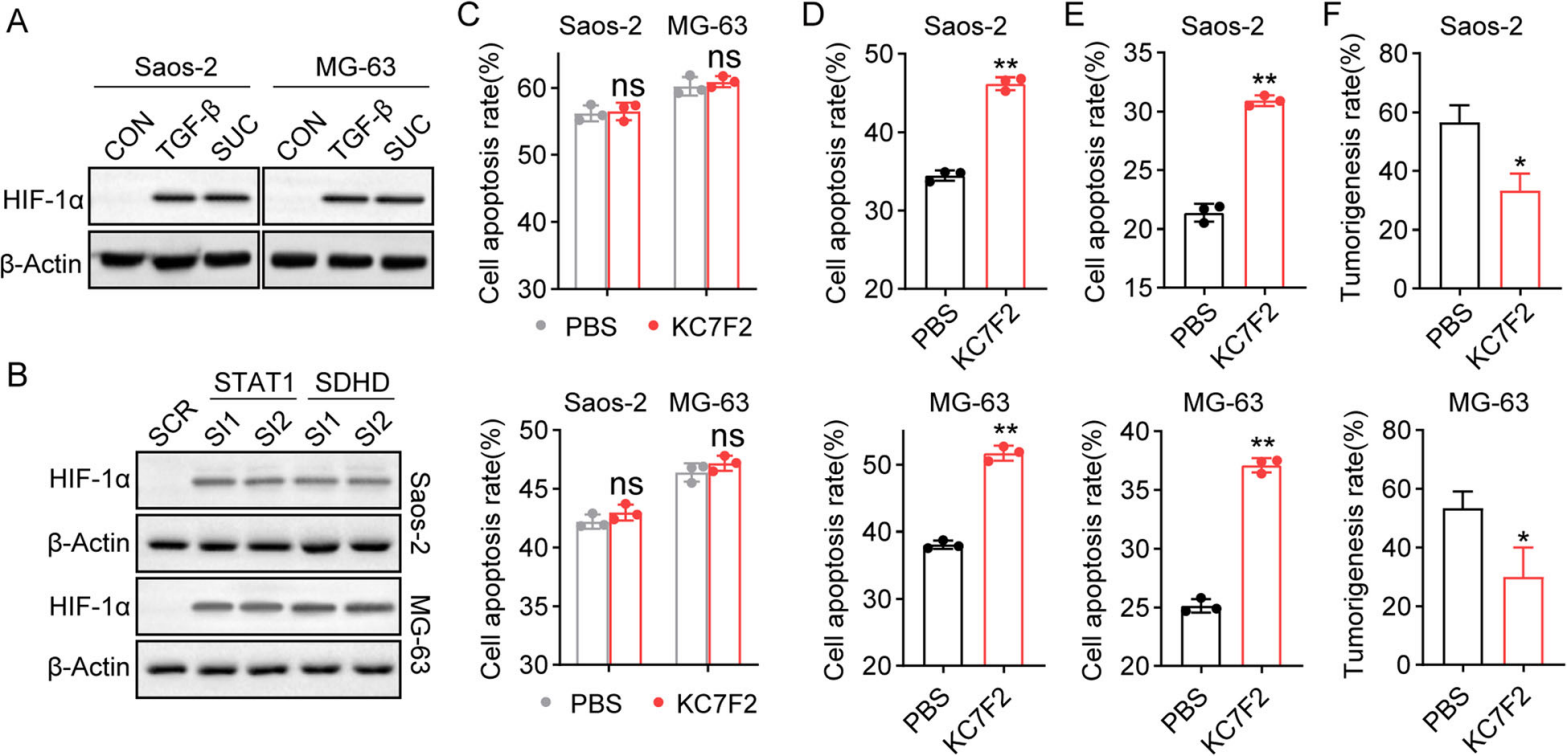
Metabolic succinate facilitated osteosarcoma chemo-resistance through an HIF1α dependent manner. **A** The expression of HIF1α in Saos-2 and MG-63 cells after treatment with TGF-β (50 ng/ml) or succinate (20 mM) was determined by western blot. **B** The expression of HIF1α in shSTAT1- or **s**hSDHD-Saos-2/MG-63 cells was determined by western blot. **C** Saos-2 and MG-63 cells were treated with MTX (75 mM)/CIS (40 μM) plus KC7F2 (20 μM). Cell apoptosis was determined by flow cytometry. **D** Saos-2 and MG-63 cells were treated with MTX (75 mM) in the presence of TGF-β (50 ng/ml) plus KC7F2 (20 μM). Cell apoptosis was determined by flow cytometry. **E** The same as D except that Saos-2 and MG-63 cells were treated with CIS (40 μM) in the presence of TGF-β (50 ng/ml) plus KC7F2 (20 μM). **F** Tumorigenicity of Saos-2 and MG-63 cells after treatment with TGF-β (20 μg/kg) plus KC7F2 (10 mg/kg). SUC, succinate; MTX, methotrexate; CIS, cisplatin

Compelling findings suggest that succinate serves as a crucial oncometabolite by suppressing PHDs, which target HIF1α for proteasomal degradation or glycolysis [25]. Hence, we sought to investigate whether TGF-β could mediate HIF1α upregulation by controlling SDH metabolism. TGF-β or succinate treatment promoted the expression of HIF1α in Saos-2/MG-63 cells (Fig. 4A). Silencing STAT1 or SDHD also contributed to the elevated expression of HIF1α (Fig. 4B), indicating that TGF-β could upregulate HIF1α in an SDH-dependent manner. Next, we used the HIF1α inhibitor KC7F2 to treat Saos-2/MG-63 cells for cytotoxicity analysis, and no obvious cytotoxicity was observed in bulk Saos-2/MG-63 cells (Fig. 4C). However, blockade of HIF1α efficiently reversed the drug resistance caused by TGF-β (Fig. 4D and E), indicating that TGF-β promoted chemoresistance through HIF1α. More importantly, our previous results indicated that TGF-β strengthened the tumorigenesis capability of osteosarcoma cells (Fig. 1F), and HIF1α has been proven to be associated with tumor stemness regulation. Herein, we further suppressed HIF1α and then treated Saos-2/MG-63 cells with TGF-β. Intriguingly, blockade of HIF1α efficiently weakened the tumorigenesis of Saos-2 and MG-63 cells in vivo (Fig. 4F), demonstrating that TGF-β could further promote osteosarcoma stemness through HIF1α. Together, these results suggested that TGF-β suppresses SDH activity to upregulate HIF1α, resulting in chemoresistance in osteosarcoma.

### Blockade of HIF1α improved the outcome of chemotherapy in osteosarcoma

**Fig. 5:**
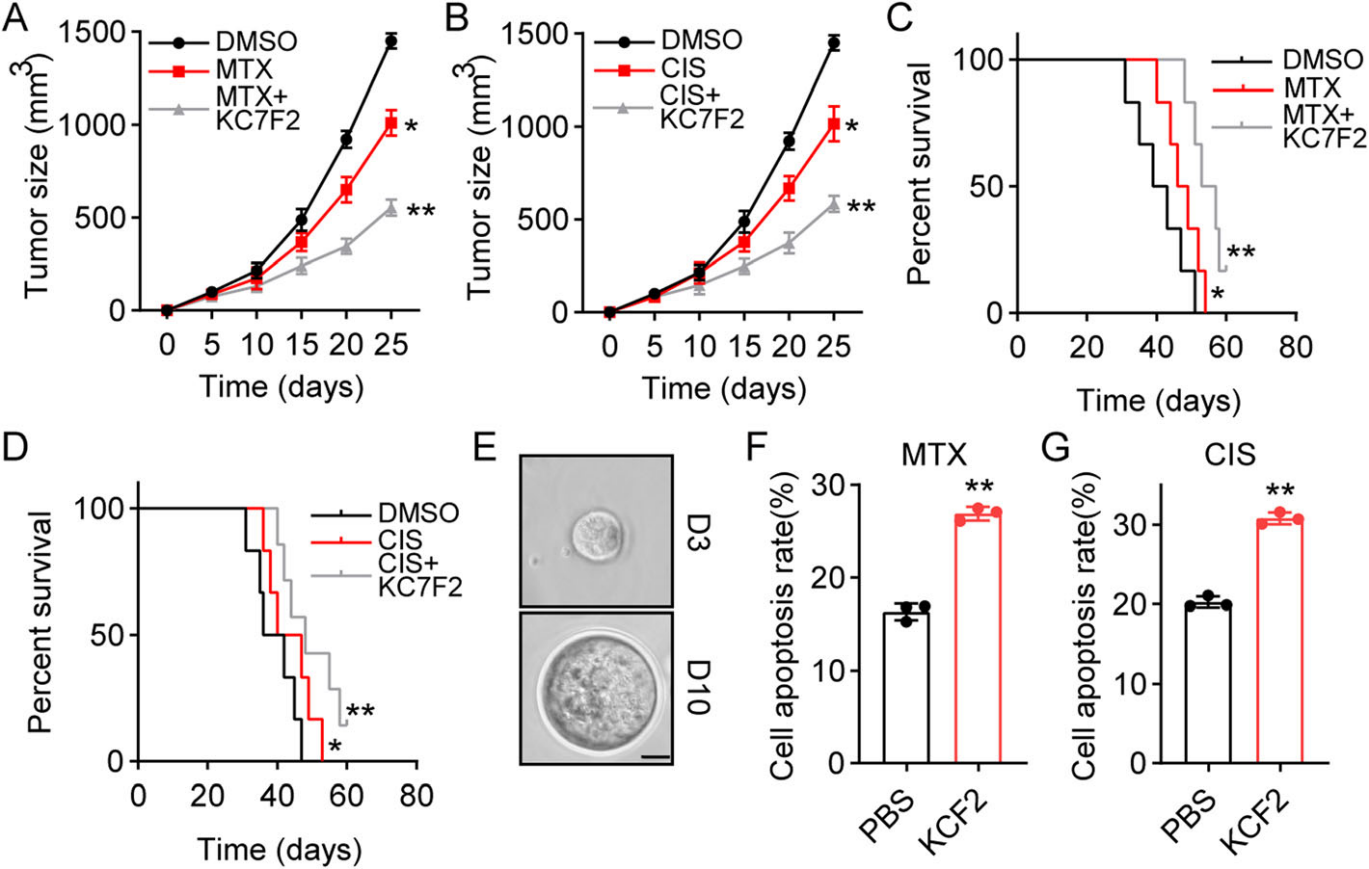
Blockade of HIF1α improved outcome of chemotherapy in osteosarcoma. **A** The tumor growth of the Saos-2 tumor was detected after treatment with DMSO, MTX (5 mg/kg), KC7F2 (10 mg/kg) and MTX combined with KC7F2. **B** The tumor growth of the Saos-2 tumor was detected after treatment with DMSO, CIS (1 mg/kg), KC7F2 (10 mg/kg) and CIS combined with KC7F2. **C** The survival time of the Saos-2 tumor was detected after treatment with DMSO, MTX (5 mg/kg), KC7F2 (10 mg/kg) and MTX combined with KC7F2. **D** The survival time of the Saos-2 tumor was detected after treatment with DMSO, CIS (1 mg/kg), KC7F2 (10 mg/kg) and CIS combined with KC7F2. **E** Osteosarcoma cells from chemo-resistant patients were cultured in 3D matrix gel. Colony size was indicated after 3 (D3) and 10 days (D10). The scale bar is 10 μm. **F** Osteosarcoma cells from chemo-resistant patients were cultured in 3D matrix gel and treated with MTX (75 mM) plus KC7F2 (20 μM). Cell apoptosis was determined by flow cytometry. **G** The same as F except that osteosarcoma cells were treated with CIS (40 μM) plus KC7F2 (20 μM). MTX, methotrexate; CIS, cisplatin

Given the crucial role of HIF1α in tumorigenesis and chemoresistance, it might be feasible to target HIF1α to improve the outcome of chemotherapy in osteosarcoma. To assess our hypothesis, Saos-2 cells were subcutaneously injected into NOD-SCID mice, followed by treatment with the HIF1α inhibitor KC7F2 and MTX/CIS. Intriguingly, the addition of KC7F2 significantly strengthened the anticancer effects of MTX (Fig. 5A) and CIS (Fig. 5B), although no TGF-β was used to treat the tumor-bearing mice. We hypothesized that osteosarcoma cells can produce TGF-β in an autocrine manner, thereby resulting in the activation of the HIF1α pathway. Consistently, KC7F2 treatment also significantly prolonged the survival time of Saos-2-bearing mice (Fig. 5C and D), indicating that suppression of HIF1α can efficiently improve the anticancer effects of chemotherapy. To further evaluate the anticancer effects of KC7F2, we isolated tumor cells from chemoresistant patients and seeded those tumor cells into matrix gels. Tumor cells isolated from patient #2 succeeded in generating spherical colonies and revealed proliferative phenotypes in 2 months (Fig. 5E). Here, we treated colonies with MTX, CIS and KC7F2 in matrix gels. As anticipated, slight cytotoxicity was observed in the MTX or CIS treatment group, which might have been due to the tumor cells being isolated from chemoresistant tumor tissue. However, the addition of KC7F2 significantly strengthened the cytotoxicity of MTX and CIS (Fig. 5F and G), indicating the potential anticancer effects of HIF1α inhibitors in the clinic. Together, these results suggested that blockade of HIF1α can improve the outcome of chemotherapy, which describes a novel strategy in clinical osteosarcoma treatment.

## Discussion

TGF-β is a pleiotropic cytokine that has a dual action in tumor development. Activation of the TGF-β pathway results in a variety of gene responses, which modulate cell cycle arrest and apoptosis in early-stage cancer, as well as metastasis and angiogenesis in advanced cancer [26]. A growing body of research has indicated that TGF-β is also linked to chemoresistance and that the combined application of drugs and TGF-β inhibitors leads to remarkable effects in several tumor types [10, 27]. However, our current understanding of TGF-β-induced drug resistance is incomplete. Given its role in gene expression and cell differentiation, cellular metabolism is emerging as a key player in tumor initiation and progression [28]. Here, our study provided evidence of the contribution of TGF-β to glucose metabolism in tumor cells, thus exacerbating drug resistance in osteosarcoma.

Variation in energetic routes is a hallmark of tumor cells and is characterized by enhancement of glucose uptake, glycolysis hyperactivation, reduction in oxidative phosphorylation, and lactate accumulation [29]. The altered cellular metabolism not only leads to an abundance of energy but also gives rise to metabolic intermediates that exert important effects in supporting tumor proliferation, metastasis, and even chemoresistance [23]. In a study of breast cancer, elevated expression of lactate dehydrogenase, which converts pyruvate into lactate, was proven to mediate trastuzumab resistance [30]. Shi et al. reported that knockdown of pyruvate kinase M2 (PKM2), the final rate-limiting enzyme of the glycolytic pathway, caused the accumulation of docetaxel in lung cancer cells and synergistically strengthened the efficiency of chemotherapy in mice [31]. In addition to enzymes in the control of glycolysis, glucose transporters also contribute to drug resistance [32]. Here, our study provided a quantitative analysis of metabolite levels and observed increased glucose consumption and lactate generation in TGF-β-treated osteosarcoma cells. Importantly, we demonstrated that TGF-β downregulated SDH expression in osteosarcoma cells, thus leading to the collection of succinate and conversion to the glycolysis pathway. Previous research revealed that TGF-β can control SDH expression through transcriptional and posttranslational regulation of STAT1 [33, 34]. Consistently, we found that TGF-β attenuated STAT1 phosphorylation to suppress SDH in osteosarcoma cells. A decrease in SDH expression in osteosarcoma cells caused the accumulation of succinate. Succinate is regarded as a crucial oncometabolite that functions by enhancing angiogenesis and suppressing histone and DNA demethylases [35, 36].. Our results indicated that succinate treatment cause a rise in HIF1α in osteosarcoma cells. Hypoxia is probably the most pervasive condition within the tumor tissue. Tumor cells overcome low oxygen tension by activating several pathways, among which HIF1α signaling plays a vital role in cancer progression. There is increasing evidence that HIF1α mediates tumor metastasis, angiogenesis and the development of resistance to various therapeutic modalities [37]. Sowa et al. reported that HIF-1 facilitated drug resistance in lung adenocarcinoma in part due to the induction of carbonic anhydrase IX (CAIX) [38]. In another study of bladder cancer, cisplatin-resistant cells exhibited higher levels of HIF1α, which was correlated with increased expression of MDR1 encoding the multidrug efflux pump P-glycoprotein (P-gp) [39]. Here, we demonstrated that HIF1α upregulation resulting from elevated succinate exerted regulatory effects on the glycolytic state and chemotherapy resistance of osteosarcoma cells.

## Conclusion

Our study indicated that TGF-β exhibited an inhibitory effect on SDH expression in osteosarcoma cells, thereby exhibiting a central function in tumor metabolism rerouting and subsequent drug resistance. Chemotherapeutic agents in combination with a HIF-1α inhibitor significantly abrogated TGF-β-mediated chemoresistance and enhanced the curative effects, which revealed a potentially promising method for combating osteosarcoma.

## Data Availability

The anonymized data used and/or analyzed during the current study are available from the corresponding author on reasonable request.

## Abbreviations

TGF-β: Transforming growth factor-β
SDH: Succinate dehydrogenase
HIF-1α: Hypoxia-inducible factor 1α
MTX: Methotrexate
CIS: Cisplatin
EMT: Epithelial-mesenchymal transition
CAIX: Carbonic anhydrase IX
P-gp: P-glycoprotein
PKM2: Pyruvate kinase M2

## References

[1] Ritter J, Bielack SS (2010). Osteosarcoma. Ann Oncol.

[2] Lilienthal I, Herold N (2020). Targeting molecular mechanisms underlying treatment efficacy and resistance in osteosarcoma: a review of current and future strategies. Int J Mol Sci.

[3] Harrison DJ, Geller DS, Gill JD, Lewis VO, Gorlick R (2018). Current and future therapeutic approaches for osteosarcoma. Expert Rev Anticancer Ther.

[4] Isakoff MS, Bielack SS, Meltzer P, Gorlick R (2015). Osteosarcoma: current treatment and a collaborative pathway to success. J Clin Oncol.

[5] Wu Q, Yang Z, Nie Y, Shi Y, Fan D (2014). Multi-drug resistance in cancer chemotherapeutics: mechanisms and lab approaches. Cancer Lett.

[6] Pan C, Wang X, Shi K, Zheng Y, Li J, Chen Y, Jin L, Pan Z (2016). MiR-122 reverses the doxorubicin-resistance in hepatocellular carcinoma cells through regulating the tumor metabolism. PLoS One.

[7] Grasso C, Jansen G, Giovannetti E (2017). Drug resistance in pancreatic cancer: impact of altered energy metabolism. Crit Rev Oncol Hematol.

[8] Varghese E, Samuel SM, Líšková A, Samec M, Kubatka P, Büsselberg D (2020). Targeting glucose metabolism to overcome resistance to anticancer chemotherapy in breast Cancer. Cancers (Basel).

[9] Min JW, Kim KI, Kim HA, Kim EK, Noh WC, Jeon HB, Cho DH, Oh JS, Park IC, Hwang SG, Kim JS (2013). INPP4B-mediated tumor resistance is associated with modulation of glucose metabolism via hexokinase 2 regulation in laryngeal cancer cells. Biochem Biophys Res Commun.

[10] Oshimori N, Oristian D, Fuchs E (2015). TGF-β promotes heterogeneity and drug resistance in squamous cell carcinoma. Cell.

[11] Cai J, Fang L, Huang Y, Li R, Xu X, Hu Z, Zhang L, Yang Y, Zhu X, Zhang H, Wu J, Huang Y, Li J, Zeng M, Song E, He Y, Zhang L, Li M (2017). Simultaneous overactivation of Wnt/β-catenin and TGFβ signalling by miR-128-3p confers chemoresistance-associated metastasis in NSCLC. Nat Commun.

[12] Katsuno Y, Meyer DS, Zhang Z, Shokat KM, Akhurst RJ, Miyazono K (2019). Chronic TGF-β exposure drives stabilized EMT, tumor stemness, and cancer drug resistance with vulnerability to bitopic mTOR inhibition. Sci Signal.

[13] Bhagyaraj E, Ahuja N, Kumar S, Tiwari D, Gupta S, Nanduri R, Gupta P (2019). TGF-β induced chemoresistance in liver cancer is modulated by xenobiotic nuclear receptor PXR. Cell Cycle.

[14] Tripathi V, Shin JH, Stuelten CH, Zhang YE (2019). TGF-β-induced alternative splicing of TAK1 promotes EMT and drug resistance. Oncogene.

[15] Akhurst RJ, Hata A (2012). Targeting the TGFβ signalling pathway in disease. Nat Rev Drug Discov.

[16] Yao Z, Fenoglio S, Gao DC, Camiolo M, Stiles B, Lindsted T, Schlederer M, Johns C, Altorki N, Mittal V, Kenner L, Sordella R (2010). TGF-beta IL-6 axis mediates selective and adaptive mechanisms of resistance to molecular targeted therapy in lung cancer. Proc Natl Acad Sci U S A.

[17] Du B, Shim JS (2016). Targeting epithelial-mesenchymal transition (EMT) to overcome drug resistance in Cancer. Molecules.

[18] Colak S, Ten Dijke P (2017). Targeting TGF-β signaling in Cancer. Trends Cancer.

[19] Duarte IF, Lamego I, Marques J, Marques MP, Blaise BJ, Gil AM (2010). Nuclear magnetic resonance (NMR) study of the effect of cisplatin on the metabolic profile of MG-63 osteosarcoma cells. J Proteome Res.

[20] Lin C, Dong J, Wei Z, Cheng KK, Li J, You S, Liu Y, Wang X, Chen Z (2020). 1H NMR-based metabolic profiles delineate the anticancer effect of vitamin C and Oxaliplatin on hepatocellular carcinoma cells. J Proteome Res.

[21] Le Belle JE, Harris NG, Williams SR, Bhakoo KK (2002). A comparison of cell and tissue extraction techniques using high-resolution 1H-NMR spectroscopy. NMR Biomed.

[22] Pickup M, Novitskiy S, Moses HL (2013). The roles of TGFβ in the tumour microenvironment. Nat Rev Cancer.

[23] Bhattacharya B, Mohd Omar MF, Soong R (2016). The Warburg effect and drug resistance. Br J Pharmacol.

[24] Luo K (2017). Signaling Cross Talk between TGF-β/Smad and Other Signaling Pathways. Cold Spring Harb Perspect Biol.

[25] Corcoran SE, O'Neill LA (2016). HIF1α and metabolic reprogramming in inflammation. J Clin Invest.

[26] Seoane J, Gomis RR (2017). TGF-β family signaling in tumor suppression and Cancer progression. Cold Spring Harb Perspect Biol.

[27] Brunen D, Willems SM, Kellner U, Midgley R, Simon I, Bernards R (2013). TGF-β: an emerging player in drug resistance. Cell Cycle.

[28] DeBerardinis RJ, Chandel NS (2016). Fundamentals of cancer metabolism. Sci Adv.

[29] Pavlova NN, Thompson CB (2016). The emerging hallmarks of Cancer metabolism. Cell Metab.

[30] Zhao Y, Liu H, Liu Z, Ding Y, Ledoux SP, Wilson GL (2011). Overcoming trastuzumab resistance in breast cancer by targeting dysregulated glucose metabolism. Cancer Res.

[31] Shi HS, Li D, Zhang J, Wang YS, Yang L, Zhang HL, Wang XH, Mu B, Wang W, Ma Y, Guo FC, Wei YQ (2010). Silencing of pkm2 increases the efficacy of docetaxel in human lung cancer xenografts in mice. Cancer Sci.

[32] Le Calvé B, Rynkowski M, Le Mercier M, Bruyère C, Lonez C, Gras T (2010). Long-term in vitro treatment of human glioblastoma cells with temozolomide increases resistance in vivo through up-regulation of GLUT transporter and aldo-keto reductase enzyme AKR1C expression. Neoplasia.

[33] Zhou X, Zöller T, Krieglstein K, Spittau B (2015). TGFβ1 inhibits IFNγ-mediated microglia activation and protects mDA neurons from IFNγ-driven neurotoxicity. J Neurochem.

[34] Reardon C, McKay DM (2007). TGF-beta suppresses IFN-gamma-STAT1-dependent gene transcription by enhancing STAT1-PIAS1 interactions in epithelia but not monocytes/macrophages. J Immunol.

[35] Mu X, Zhao T, Xu C, Shi W, Geng B, Shen J (2017). Oncometabolite succinate promotes angiogenesis by upregulating VEGF expression through GPR91-mediated STAT3 and ERK activation. Oncotarget.

[36] Xiao M, Yang H, Xu W, Ma S, Lin H, Zhu H, Liu L, Liu Y, Yang C, Xu Y, Zhao S, Ye D, Xiong Y, Guan KL (2012). Inhibition of α-KG-dependent histone and DNA demethylases by fumarate and succinate that are accumulated in mutations of FH and SDH tumor suppressors. Genes Dev.

[37] Fallah J, Rini BI (2019). HIF inhibitors: status of current clinical development. Curr Oncol Rep.

[38] Sowa T, Menju T, Chen-Yoshikawa TF, Takahashi K, Nishikawa S, Nakanishi T, Shikuma K, Motoyama H, Hijiya K, Aoyama A, Sato T, Sonobe M, Harada H, Date H (2017). Hypoxia-inducible factor 1 promotes chemoresistance of lung cancer by inducing carbonic anhydrase IX expression. Cancer Med.

[39] Sun Y, Guan Z, Liang L, Cheng Y, Zhou J, Li J (2016). HIF-1α/MDR1 pathway confers chemoresistance to cisplatin in bladder cancer. Oncol Rep.

